# Multiple Daily Rounds of Theta-Burst Stimulation for Tinnitus: Preliminary Results

**DOI:** 10.3390/medicina57080743

**Published:** 2021-07-23

**Authors:** Seok-Min Hong, Sung-Kyun Kim, Moo-Young Seo, Suk-Yun Kang

**Affiliations:** 1 Department of Otorhinolaryngology-Head and Neck Surgery, Dongtan Sacred Heart Hospital, Hallym University College of Medicine, Hwaseong 18450, Korea; thecell@hallym.or.kr (S.-M.H.); newearera@hallym.or.kr (S.-K.K.); 2 Department of Neurology, Dongtan Sacred Heart Hospital, Hallym University College of Medicine, Hwaseong 18450, Korea; zerozz2@hallym.or.kr

**Keywords:** tinnitus, repetitive transcranial magnetic stimulation, continuous theta-burst stimulation, multiple rounds, safety, Tinnitus Handicap Inventory, minimum masking level, residual inhibition

## Abstract

*Background and Objectives*: Tinnitus is a condition that negatively affects the quality of life and is difficult to treat. Theta burst stimulation (TBS), a new method of repetitive transcranial magnetic stimulation (rTMS), is a promising treatment approach because it shows stronger and more prolonged effects in a shorter time of stimulation than other rTMS protocols. However, the therapeutic effect of TBS for tinnitus was inconsistent. We hypothesized that more stimulation would be more effective. Therefore, this study aimed to explore the safety and effectiveness of multiple daily rounds of TBS over five consecutive days. *Materials and Methods*: The continuous TBS (cTBS) protocol is 300 pulses/day, but we applied 8 sessions of 300 pulses in a day (total 2400 pulses/day). A total of 15 patients with tinnitus were randomly assigned to treatment and sham groups. Outcome measurements were taken three times: before and after 5-day of stimulation; at a 1–3 month follow-up visit. Outcome measurements were the degree of annoyance due to ear fullness, duration of tinnitus, visual analog scales of tinnitus for annoyance, Tinnitus Handicap Inventory, pitch, loudness, minimum masking level, and residual inhibition. *Results*: Five-day cTBS was completed without adverse events. We did not find any significant therapeutic effect in the treatment group, but we needed to be cautious to interpret our result due to the small sample size. *Conclusions*: In conclusion, multiple rounds of cTBS in a day may be safe. Further research is needed in a larger sample size to determine the effectiveness and confirm the safety.

## 1. Introduction

There is no established cure for tinnitus [1]. The prevalence is estimated to range from 10 to 25% and has increased in younger populations over the past years, possibly due to frequent exposure to leisure noise [2]. A total of 6–25% of patients with tinnitus complain of severe quality of life issues [3], including sleep, concentration, emotion, and social enjoyment [2].

The mechanism of tinnitus is poorly understood. Maladaptive neural plasticity of fronto-striatal and auditory cortical areas was suggested to be related to tinnitus [4]. Tinnitus-related activity changes occur in the auditory and non-auditory pathways in the brain. Non-auditory pathways include a consciousness-supporting network such as the anterior insula, anterior cingulate, thalamus, and amygdala [5]. Neuromodulation such as repetitive transcranial magnetic stimulation (rTMS) has been used for tinnitus treatment, but the therapeutic effects were different among studies [6,7]. rTMS is a non-invasive technique to adjust cortical excitability with repetitive magnetic pulses. High-frequency rTMS increase cortical excitability, and low-frequency rTMS decrease the excitability. In addition to depression, the range of clinical applications for neurological and psychiatric diseases such as Parkinson’s disease, cerebral infarction, and pain is expanding [8].

Theta burst stimulation (TBS), a new method of rTMS, was introduced as a very rapid method of conditioning the human brain because of just a 20–190 s stimulation period. It is a patterned stimulation of three pulse-delivery at 50 Hz repetition every 200 ms. There are two paradigms: intermittent TBS (iTBS) is a 2 s train of TBS repeated every 10 s for a total of 190 s (600 pulses) and continuous TBS (cTBS) is an unceasing 40 s train of TBS [9]. It may have advantages compared with conventional rTMS, considering shorter stimulation time, lower stimulation intensity, and prolonged effects after stimulation [10]. The therapeutic effect of TBS was published in various neurological disorders such as stroke, multiple sclerosis, and dystonia [8]. However, the few tinnitus studies conducted on TBS have yielded inconsistent results [11,12,13]. We hypothesized that more stimulation would be more effective.

This prospective, preliminary study aimed to explore whether multiple daily rounds of TBS over five consecutive days is safe and effective in tinnitus patients. Because it was a preliminary study, the primary objective was to assess its safety.

## 2. Methods

### 2.1. Patients 

Patients with unilateral or bilateral tinnitus were recruited from an otolaryngology outpatient clinic after a complete examination by 2 otolaryngologists (S.M.H. and S.K.K.) from 2014 to 2018. All participants provided written informed consent before being randomized into this study. Inclusion criteria were patients over 18 years old with subjective tinnitus for more than 2 months and who had no improvement with medication. Exclusion criteria were (1) Meniere’s diseases, conductive hearing loss, objective tinnitus, (2) a history of seizure disorder, previous symptomatic stroke, (3) surgically or traumatically implanted foreign bodies such as a pacemaker, an implanted medication pump, metal in the skull or eyes (other than dental appliances or fillings), or intracardiac lines that might pose a physical hazard during magnetic stimulation. At the baseline evaluation, handedness and depression were assessed using the Edinburgh handedness inventory [14] and Beck Depression Inventory, respectively. All subjects gave their informed consent for inclusion before they participated in the study. This study was conducted in accordance with the Declaration of Helsinki. The protocol was approved by our Institutional Review Board, the Ethics Committee of Dongtan Sacred Heart Hospital (Project identification code: 2013-109). The clinical trial identifier number was NCT02071732.

### 2.2. Study Design

This study was a randomized, double-blind, sham-controlled design. Both patients and investigators (outcomes assessors) were blinded to treatment conditions. We used computer-generated random numbers for the randomization. After enrollment, each patient was determined to the type of intervention with a random number. After random assignment (i.e., real and sham rTMS conditions), brain stimulation was applied daily over 5 consecutive days. Outcome measurements were taken 3 times, before rTMS on the 1st day, immediately after rTMS on the 5th day, and 1 to 3 months after treatment. In the sham condition, one-wing of the figure-eight in contact with the scalp was 90° tilt from tangential to the scalp [15]. Patients and the 2 otolaryngologists were blinded about which stimulation was applied, and the otolaryngologists assessed the outcome measurements.

### 2.3. rTMS Protocol

In the real rTMS group, the center of the D70^2^ coil was placed over the left temporoparietal cortex halfway between T3 and P3, per the international 10–20 system and the handle of the coil was directed upward [16]. In the sham group, the rim of the coil was positioned perpendicular to the head [15]. The resting motor threshold (RMT) was measured with the right abductor pollicis brevis muscle as the lowest stimulus intensity required to produce motor-evoked potentials of at least 50 μV in at least 5 of 10 consecutive trials. 

One session of continuous theta-burst stimulation (cTBS) involved 3 TMS pulses of 50 Hz (i.e., 20 ms between each stimulus) repeated at a 200 ms interval (i.e., 5 Hz) for 20 s at a stimulus intensity of 70% RMT. This 70% RMT was set based on previous reports, based on the equivalence of 80% active motor threshold (AMT) [17,18]. We applied 4 sessions at a 1-s interval, After 15 min, another 4 sessions with a 1-s gap between sessions, per day (2400 pulses/day), modified from previous studies [19,20]. The decision to give 300 pulses at a time followed the recommendation set by the TMS machine company. cTBS was delivered using a Magstim Super-Rapd^2^ stimulator (Magstim, Wales, UK). We adopted a 15 min break, which followed previous methodological studies that showed a dose-dependent effect [21,22].

### 2.4. Outcome Measurement

Outcome measurements were the degree of annoyance due to ear fullness, duration of tinnitus (hours/day), visual analog scales (VAS) of tinnitus for annoyance, Tinnitus Handicap Inventory (THI), pitch (Hz), loudness (dbSL), minimum masking level (MML, dbSL), and residual inhibition (RI). 

The degree of ear fullness ranged from 0 to 4: 0 = little or no interference; 1 = some interference; 2 = takes considerable effort to maintain normal activity; 3 = serious interference; 4 = unable to perform any work [23]. VAS was given on a numeric rating scale from 1 to 10: 1 = no annoyance; 10 = worst annoyance. The THI was a self-reported measure quantifying the impact of tinnitus on daily living ranging from 0 to 100: 0–16: no handicap; 18–36: mild handicap; 38–56: severe handicap; 58–100: catastrophic handicap [24]. The THI also had 3 subscales: functional, emotional, and catastrophic response subscales. Tinnitograms were obtained simultaneously as pure tone audiometry using GSI Audera (Grason-Stadler Inc, Eden Prairie, MN, USA). The frequency and loudness of tinnitus were identified by matching, respectively; when not identified in narrow-band noise, it was assessed using white noise. Assessment of tinnitus pitch was significant for systematic documentation of patients’ symptoms and monitoring the impact of interventions, and planning tinnitus treatment involving acoustic stimulation such as tinnitus maskers [25]. Although psychoacoustical characteristics of tinnitus (such as tinnitus pitch, loudness, etc.) do not appear to determine tinnitus annoyance or severity of the complaint, they may be useful markers for neural plasticity if the tonotopic representation in the central auditory system was modified after treatment [26]. As a masker noise, the narrow-band noise of the tinnitus frequency obtained from the tinnitus pitch test was used for MML measurement. If the tinnitus frequency was a narrow or wide band, wide band noise was used. About 2 s of masker noise was presented, it was measured that the lowest level of noise necessary to mask the patient’s tinnitus. The examiner increased the masker noise by 5 dB and found the minimal intensity of the masking sound that made the tinnitus inaudible. For RI measurement, the noise used to measure the minimal masking level was used. The examiner gave the patients a narrow or wide band noise 10 dB higher than the tinnitus, which lasted for 1 min and recorded the time in seconds until the tinnitus was felt again after the sound stimulation stop. Positive means the tinnitus disappeared or the tinnitus loudness was reduced.

### 2.5. Statistical Analysis

Data were expressed as mean ± standard deviation (SD). Demographics and clinical variables between the real and sham groups were compared with the Mann–Whitney, chi-square, or Fisher’s exact test, as appropriate. Continuous variables were analyzed with the Mann–Whitney test and categorical variables were analyzed with chi-square or Fisher’s exact test. A generalized linear mixed model (GLMM) approach evaluated the rTMS effect over time between the 2 groups. If there was a significant interaction between group and time, multiple pairwise comparisons were controlled using Bonferroni correction. A *p*-value less than 0.05 was considered significant. Statistical analysis was performed using IBM SPSS 24 Statistics (IBM Corp., Armonk, NY, USA).

## 3. Results

Data from 13 of 15 patients were analyzed. Two patients were excluded due to technical problems during the experiment. Demographics and clinical features between the real and sham groups are summarized in Table 1. There were no significant differences between the groups. 

**Figure 1:**
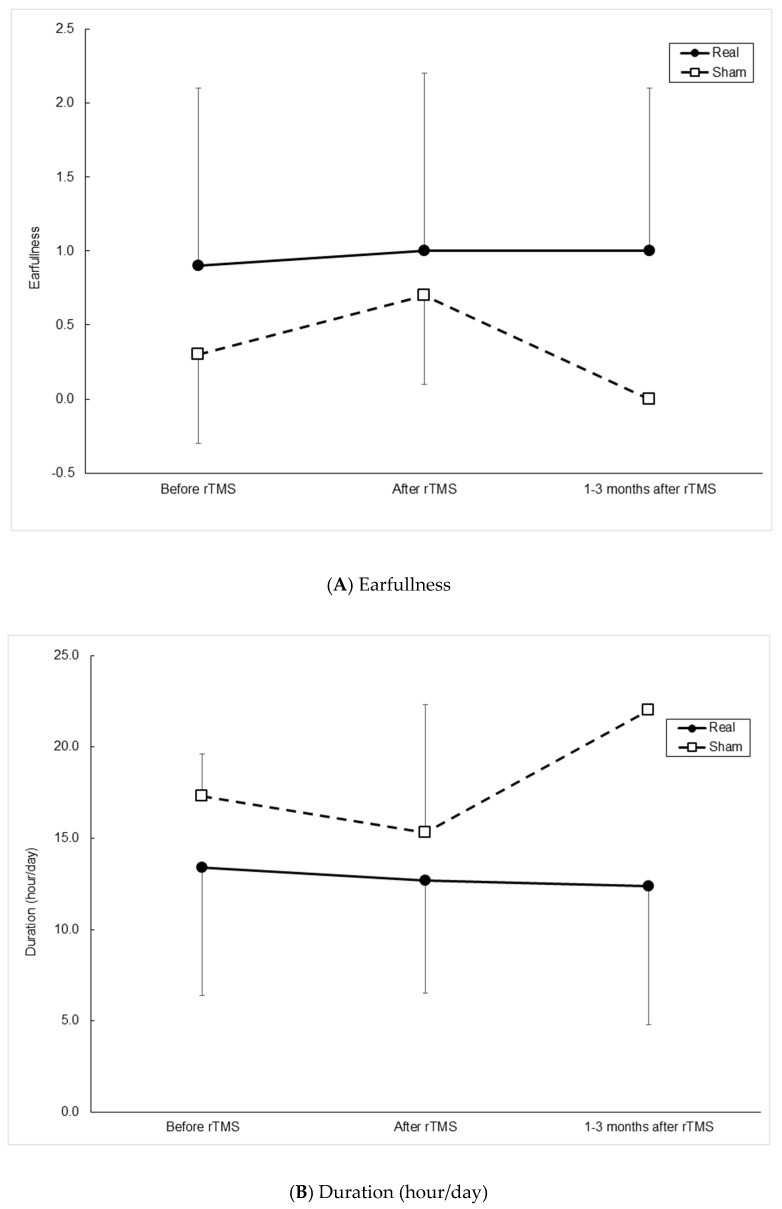

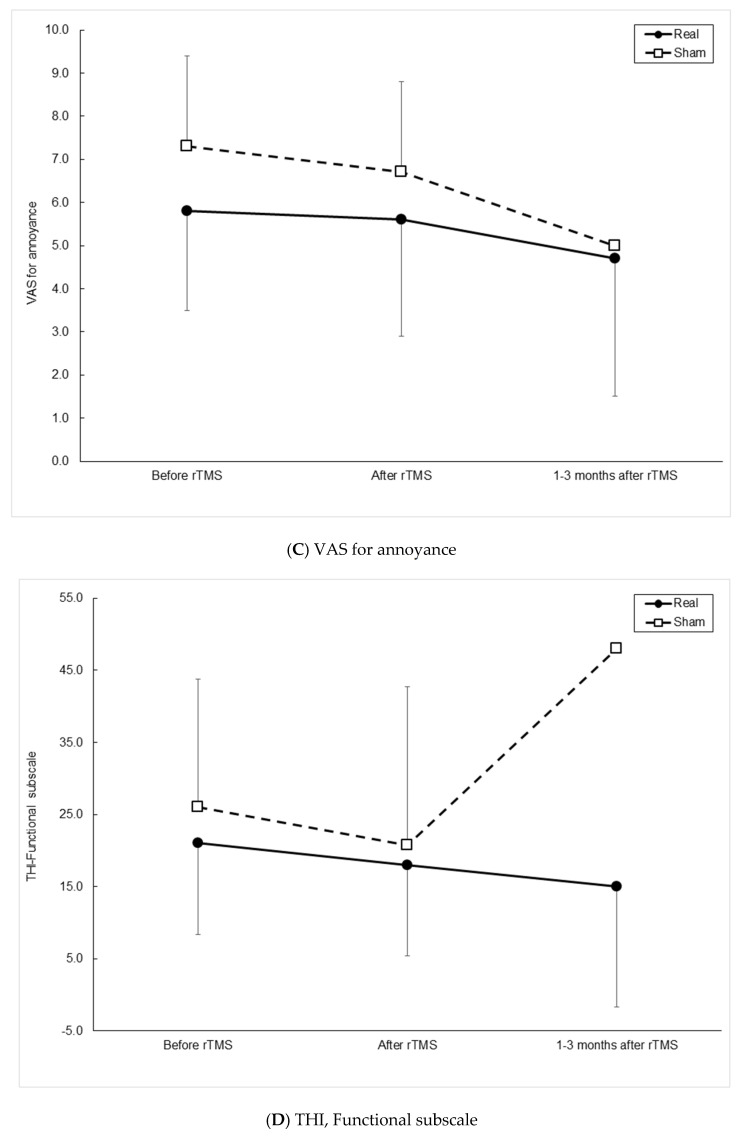

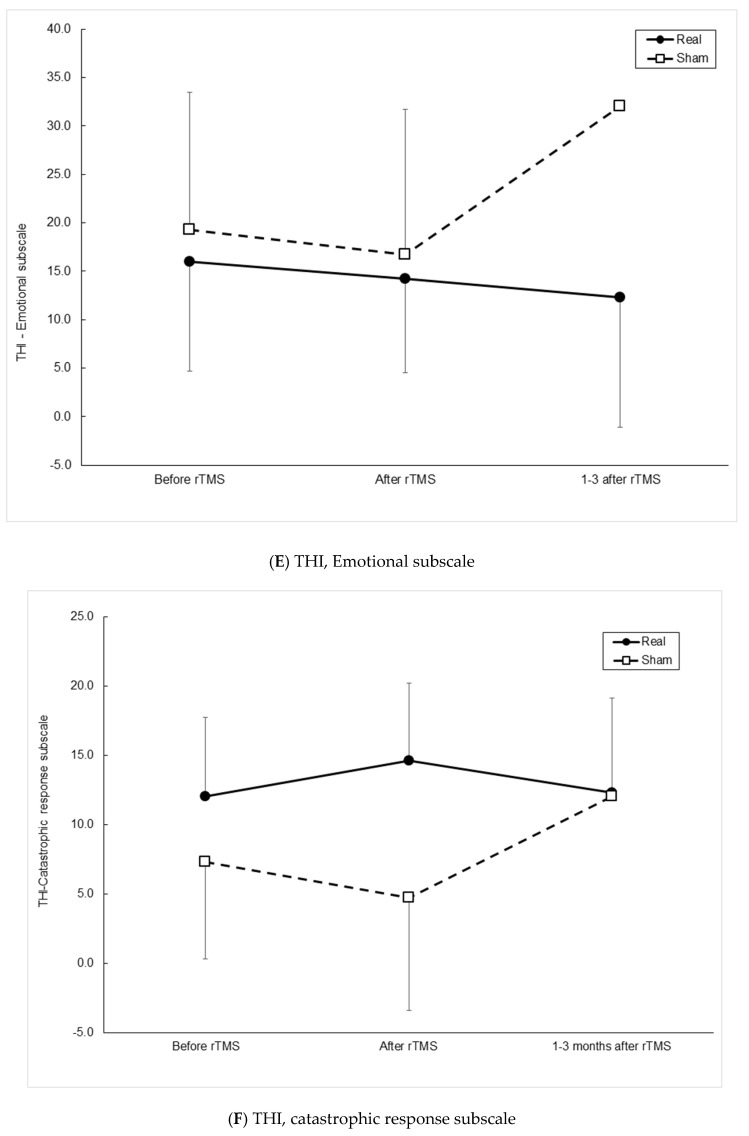

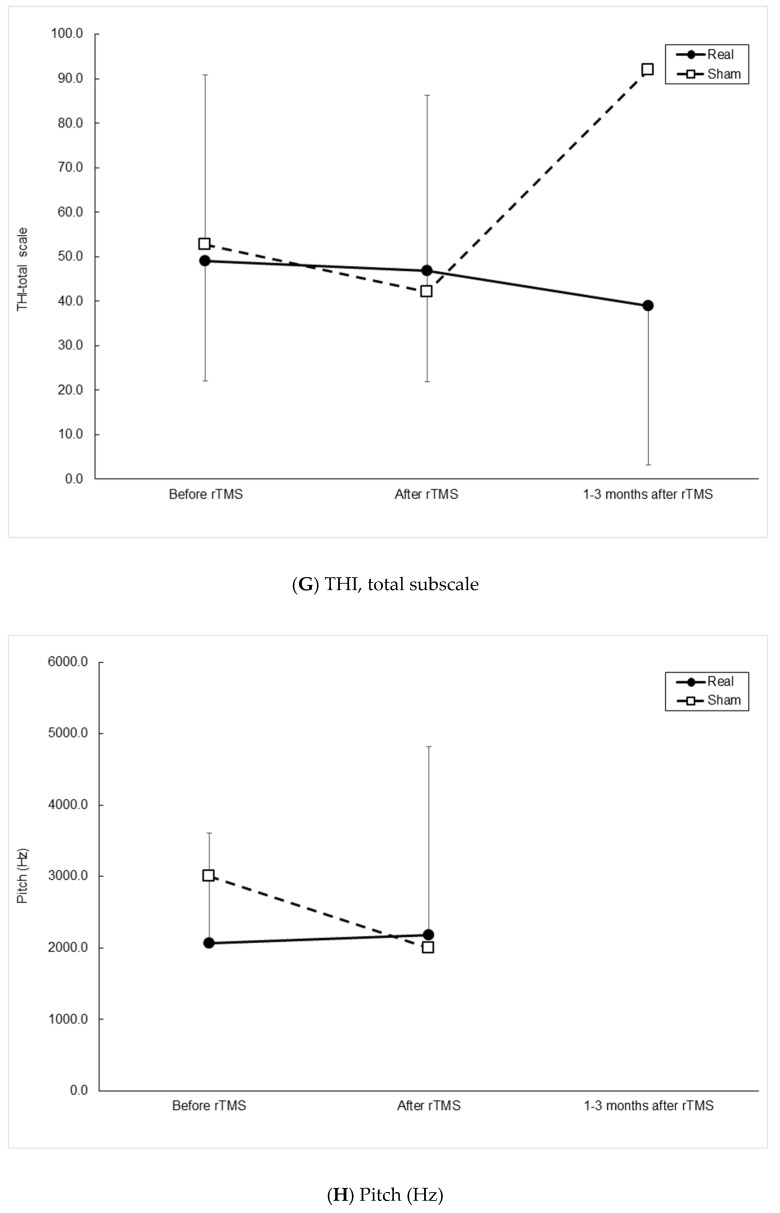

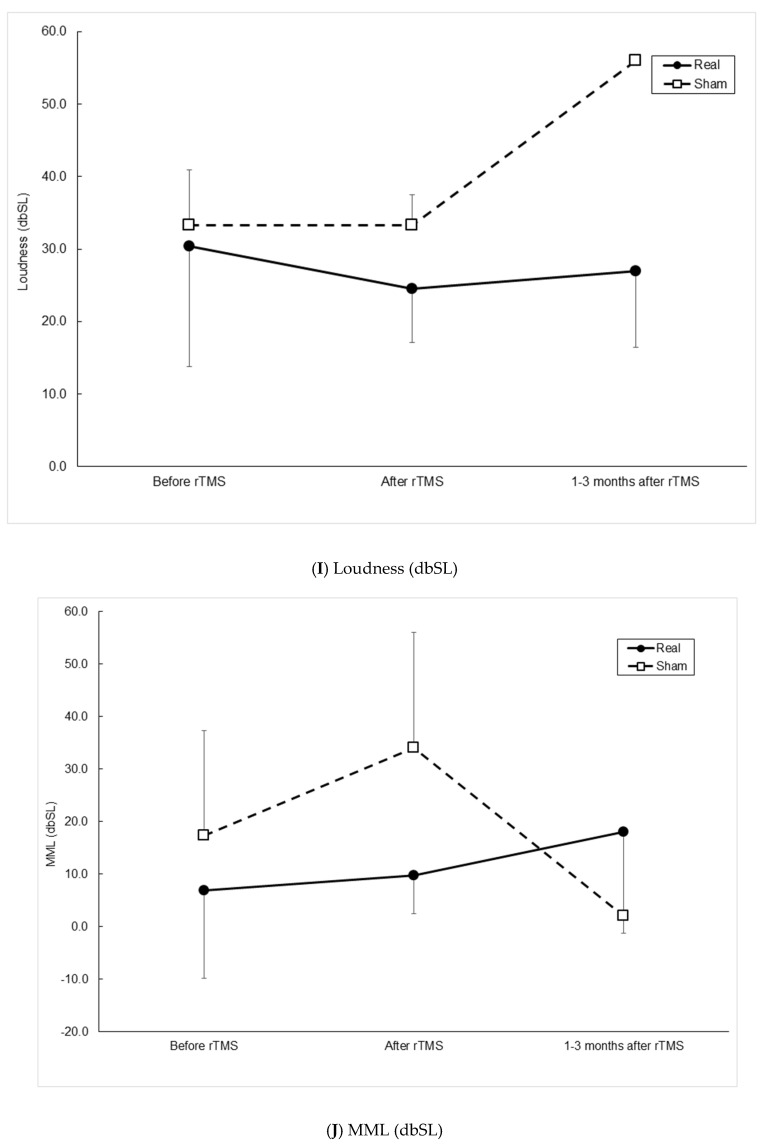

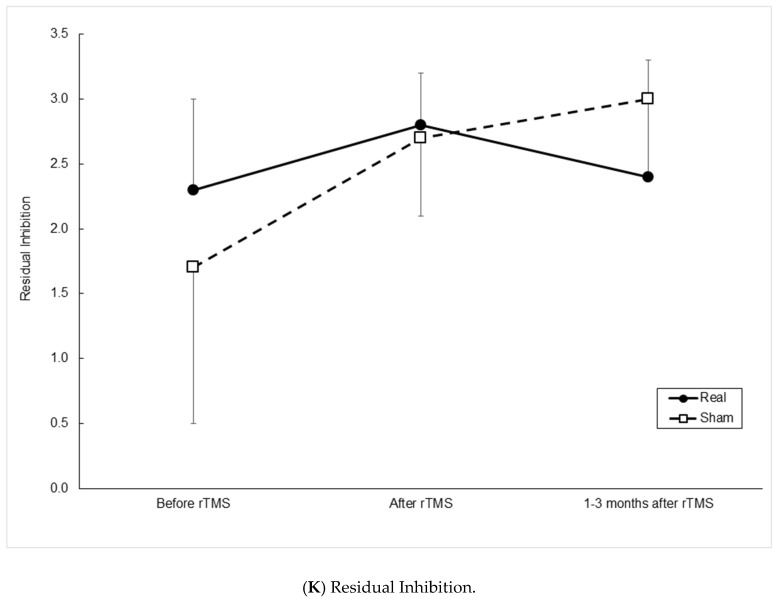
Outcome measurements between real and sham continuous theta-burst stimulation groups before brain stimulation, after brain stimulation on the 5th day, and at follow-up 1–3 months later. (**A**) The degree of annoyance of ear fullness, (**B**) duration of tinnitus, (**C**) visual analog scale (VAS) of tinnitus annoyance, (**D**) Tinnitus Handicap Inventory (THI), functional subscale, (**E**) THI, emotional subscale, (**F**) THI, catastrophic subscale, (**G**) THI, total score, (**H**) pitch, (**I**) loudness, (**J**) minimal masking level, and (**K**) residual inhibition. A generalized linear mixed model approach showed significant differences of changing pattern in VAS and MML over the three visits between two groups ((**C**), *p* = 0.002 and (**K**), *p* = 0.033, respectively). This was supposed to be due to the large changes over time in the sham group, but there were no significant differences in the mean values of VAS and MML in paired analyses in each group (all ps > 0.2). Besides, there were no significant differences in the mean values of VAS and MML between the two groups at each visit (ps > 0.2 and ps > 0.1, respectively).

Outcome measurements between the two groups over time are presented in Figure 1. At a visit 1–3 months after rTMS, 7 patients participated (6 in the real group, 1 in the sham group). GLMM analysis suggested that the pattern of changes over time in the mean values of VAS and MML were significantly different between the two groups (Figure 1C, *p* = 0.002 and Figure 1J, *p* = 0.033, respectively). The pattern of changes appeared more remarkable in the sham group. Still, there was no significant difference in VAS or MML between the two groups at each time point (ps > 0.2 and ps > 0.1, respectively). In the paired analysis (before and after rTMS, before and 1–3 months after rTMS, after rTMS, and 1–3 months after rTMS), there was no difference in VAS or MML in either group (all ps > 0.2), probably because the number of follow-up of 1–3 months patients was too small. The other outcome measurements did not show any difference between the two groups over time. 

Differences in the outcomes were calculated between before and immediately after rTMS, and between before and 1–3 months after rTMS.Statistical analysis revealed the same results as above.

In the sub-analysis of our real TMS group, there was no difference in the rTMS effect between the depression and no depression groups (Supplementary Table S1, all ps > 0.1).

## 4. Discussion

This preliminary study has shown that our cTBS protocol (2400 pulses/day) can be conducted safely without any side effects. It also suggested that the change patterns of the mean value of VAS of tinnitus for annoyance and of the mean value of MML were significantly different over time between the real and sham groups. Maybe this was due to the significant change over time in the sham group. Independent analyses between the real and sham groups showed no difference in these values, and paired comparisons in each group also showed no differences. Although the small sample size of the sham group made statistical interpretation difficult, at least in the real group, cTBS does not seem effective because there was no significant difference on paired analysis.

Our study provided evidence that a stimulation period of up to 2400 pulses/day in one brain region is a safe protocol and supports previous similar findings. For example, although the stimulation site was different, our results were in line with that reported for depression treatment by TBS, saying that TBS 1800 to 3600 pulses/day was safe [27]. 

Previously, four studies using TBS were reported (Supplementary Table S2). Three were sham-controlled [11,12,13], and the other was a comparison between TBS and high-frequency TMS intervention [28]. The stimulation site was the temporoparietal or auditory cortex in the four studies, similar to ours. The stimulation protocols were different from ours: 1200 pulses/day for 5 consecutive days was used in one sham-controlled study [12] and another comparison study [28]; the first produced negative results and the latter positive results. Another sham-controlled study used 900 pulses/day for 10 consecutive business days, and this approach was reported to be effective [11]. The last one seemed similar to ours [13] because they used 2400 pulses/day, but several things were different. First, we applied 2400 pulses only to the left hemisphere, but the previous study stimulated both hemispheres, and 1200 pulses were delivered to each hemisphere. Second, the method of determining the intensity of stimulation was different. We used the RMT to determine the stimulation intensity at each visit instead of AMT because we thought that we could set the stimulation intensity more stably. Third, because a total of 2400 pulses were given to one hemisphere, we were concerned about safety, thus we divided it into 300 pulses per session. According to the previous studies, stimulation periods may also be significant. The TBS seemed to be effective for tinnitus in a more extended stimulation period of more than one week. We stimulated for only one weekday. Outcome measurements were different among the four studies and ours. Different outcome variables could lead to different results among the studies, but we extensively assessed the effect of the treatment using a number of measurements.

We used 2400 pulses/day for 5 consecutive days because we presumed the long duration of brain stimulation would be more effective over a more extended period, based on previous studies [19,20]. However, since contradictory results have also been reported, we should be careful about choosing long-duration TBS at this time [29]. 

More studies may be needed about which area to stimulate. We chose the left temporoparietal cortex irrespective of the tinnitus site because most previous studies have stimulated the left temporal cortex. However, some previous conventional rTMS studies stimulated different brain areas: the bilateral auditory cortex [30], left frontal and temporal area [31], and the temporal cortex ipsilateral or contralateral to the tinnitus symptoms [16].

Our study has some limitations. First of all, because of the small sample size and loss to follow-up, it was not easy to interpret the results and conclude. Therefore, it was just a preliminary study, and I think we should be careful about interpreting the treatment effect. The small sample size would have led to a type II error due to the study’s low power. Besides, only one participant could be assessed at 1–3 months after treatment in the sham group. Second, comorbidities such as depression may have affected the results. Depression is common in patients with tinnitus, ranging from 14% to 80% [32], higher than in the general population [33]. Mutual interactions between depression and tinnitus and a shared neural network have been suggested [32,33]. However, a small number of rTMS studies have investigated depressive symptoms. We did not find any evidence of a depression effect for our results because there was no difference of rTMS effect between the depression and no depression groups in the sub-analysis of our real TMS group (Supplementary Table S1). Third, we localized the left temporoparietal cortex by using the international 10–20 system instead of a neuronavigation system based on previous publications, but this may be suboptimal for the localization compared with the neuronavigation system. However, It was unlikely to affect our results because no difference in outcomes between using the 10–20 system and using neuronavigation systems was reported [34]. Forth, because a previous study said that conventional facilitatory iTBS converted into inhibitory when it was applied for twice as long, while the normally inhibitory cTBS became facilitatory when the stimulation duration was doubled [29], our inhibitory protocol might change as an excitatory signal during the stimulation. However, as mentioned above, because the accumulation effect of TBS was also reported in other studies, it seems to be remained to be confirmed. Fifth, we had difficulty seeing patients strictly at 1 month or 3 months after treatment. However, the lasting effect of TBS is short, thus the broad periodic follow-up of 1 to 3 months can cause different results for the cTBS effect. Sixth, from a safety point of view, although we closely checked participants’ conditions such as hearing problems or mood/cognitive changes during the study, we did not measure them using questionnaires or functional image studies. However, we think there was no problem with them in our study because our outcome measurements were to assess many different aspects of tinnitus, and some of the negative results were for the hearing changes or mood changes related to tinnitus.

## 5. Conclusions

In conclusion, a long duration of TBS can be applied safely. TBS is a neuromodulation approach to treating tinnitus with many advantages. However, further research should be conducted on TBS location and protocols in tinnitus to determine its effectiveness.

## Figures and Tables

**Table 1:** Characteristics of patients with tinnitus.

	Real (*n* = 10)	Sham (*n* = 3)	*p*-Value
Age, years	55.1 ± 11.6	62.0 ± 16.7	0.469
Women, *n* (%)	3 (30.0)	2 (66.7)	0.315
Duration of disease, months	28.1 ± 39.6	81.3 ± 67.0	0.217
BDI	14.1 ± 10.4	25.7 ± 15.9	0.287
Tinnitus location, *n* (%)			0.315
Right	-	-	
Left	3 (30.0)	2 (66.7)	
Bilateral/in the head	7 (70.0)	1 (33.3)	
EHI	97.5 ± 4.2	73.3 ± 46.2	0.811
Stimulus intensity (%)	43.6 ± 5.2	37.7 ± 10.8	0.217

BDI, Beck Depression Inventory; EHI, Edinburgh Handedness Inventory; RMT, Resting Motor Threshold.

## Data Availability

Data is contained within the article or supplementary material.

## Supplementary Materials

The following are available online at https://www.mdpi.com/article/10.3390/medicina57080743/s1, Table S1: Subanalysis the rTMS effect between the depression and no depression groups in real TMS group, Table S2: Characteristics of previous published continuous TBS studies.
