# Supplementary material for: Multiple Daily Rounds of Theta-Burst Stimulation for Tinnitus: Preliminary Results
Source: Medicina (Kaunas). 2021 Jul 23;57(8):743. doi: 10.3390/medicina57080743 (PMC8401076; doi:10.3390/medicina57080743)
Supplement: Supplementary file 1 [file medicina-57-00743-s001.zip › medicina-1301339-supplementary.pdf]

Supplementary Table S1. Subanalysis the rTMS effect between the depression and no depression groups in real TMS group

Outcome measurement	No depression (n = 5)*			Depression (n = 5)*			p-value
	Before	After	1-3 months after	Before	After	1-3 months after	
Ear fullness	0.8±1.3	0.8±1.3	0.3±0.6	1.0±1.2	1.2±1.3	1.7±1.2	0.683
Duration (hours/day)	10.6±6.1	10.6±6.1	9.5±8.0	16.2±7.4	14.8±6.1	15.3±7.2	0.183
VAS for annoyance	4.8±2.8	4.2±3.0	2.3±2.3	6.8±1.3	7.0±1.4	7.0±2.0	0.367
THI-F	18.0±12.7	13.2±10.3	3.3±1.2	24.0±13.1	22.8±13.9	26.7±17.0	0.802
THI-E	10.8±10.8	7.6±7.4	1.3±2.3	21.2±10.2	20.8±6.9	23.3±9.0	0.874
THI-C	8.8±2.3	9.6±2.6	8.0±5.3	15.2±6.4	19.6±0.9	16.7±5.8	0.490
THI-T	37.6±23.5	30.4±17.1	12.7±2.3	60.4±27.5	63.2±20.9	66.7±30.1	0.708
Pitch (Hz)**	1700.0±1708.4	1250.0±750.0	1083.3±878.0	3000.0±0.0	4500.0±4949.7	-	0.297
Loudness (dbSL)	35.2±21.0	25.2±-8.4	32.7±7.0	25.6±11.1	24.0±7.1	21.3±11.7	0.674
MML (dbSL)	0.8±14.3	8.4±8.0	7.3±16.7	14.5±18.4	11.5±7.0	34.0±8.5	0.497
Residual inhibition	2.0±0.7	2.8±0.4	2.3±1.2	2.8±0.4	2.8±0.5	2.5±0.7	0.291

Patients with a Beck depression inventory score of 16 or higher were considered as having depression [1,2].

*The number of patients 1-3 months after rTMS in each group was three.

**We failed to collect the data of pitch in some depressive patients. The number of depressive patients before, after, and 1-3 months after rTMS was 2, 2, and 0, respectively.

References:

1. Jo SA, Park MH, Jo I, Ryu SH, Han C. Usefulness of Beck Depression Inventory in the Korean elderly population. *Int J Geriatr Psychiatry.* (2007) 22:218-223. doi: 10.1002/gps.16642.

2. Park K, Jaekal E, Yoon S, Lee SH, Choi KH. Diagnostic Utility and Psychometric Properties of the Beck Depression Inventory-II Among Korean Adults.Font Pscyhol. (2020) 10:2934. doi: 10.3389/fpsyg.2019.02934.

Supplementary Table S2. Characteristics of previous published continuous TBS studies

Study (reference number)	Study design	Number of participants	Stimulation site	Stimulation intensity	Number and frequency of sessions	Follow-up after treatment	Results
Chung et al., 2012 [11]	Randomized, sham-controlled	22	Left auditory cortex	80% RMT	10 working days; 900 pulses/day	1 month	effective
Godbehere et al., 2019 [12]	Randomized, sham-controlled	40	Contralateral Temporal-parietal (contralateral to the affected ear or left in bilateral tinnitus)	80% AMT	5 consecutive days; 600 pulses/day	2 and 4 weeks	not effective
Plewnia et al., 2012 [13]	Randomized, sham-controlled (3-armed trial)	48	The secondary auditory cortex or the temporoparietal association cortex	80% AMT	5 working days for 4 weeks; 1200 pulses/day for each hemisphere (15 minutes after the first 600 pulses for each hemisphere, a second pair of cTBS was given)	2, 4, and 12 weeks	not effective
Forogh et al., 2013 [28]	Randomized, controlled with high-frequency rTMS (HF-rTMS)	55	Each auditory cortex (cTBS) and the left auditory cortex (HF-rTMS)	80% RMT	Weekly for four consecutive weeks; 600 pulses; 1200 pulses/day for each hemisphere in cTBS group (15 minutes after the first 600 pulses for each hemisphere, a second pair of cTBS was given); 1000	1 month	More effective in cTBS group than in HF-rTMS group

					pulses/day for 10 minutes in HF-rTMS group		
